# Supplementary material for: Auto-accelerated dehydrogenation of alkane assisted by in-situ formed olefins over boron nitride under aerobic conditions
Source: Nat Commun. 2023 Jan 5;14:73. doi: 10.1038/s41467-022-35776-3 (PMC9814760; doi:10.1038/s41467-022-35776-3)
Supplement: Supplementary file 1 — Supplementary Information [file 41467_2022_35776_MOESM1_ESM.pdf]

# Supplementary Information

# Auto-accelerated dehydrogenation of alkane assisted by in-situ formed olefins over boron nitride under aerobic conditions

Zhankai Liu, Ziyi Liu, Jie Fan, Wen-Duo Lu, Fan Wu, Bin Gao, Jian Sheng, Bin Qiu, Dongqi Wang, An-Hui Lu*

State Key Laboratory of Fine Chemicals, Liaoning Key Laboratory for Catalytic Conversion of Carbon Resources, School of Chemical Engineering, Dalian University of Technology, Dalian 116024, Liaoning, China

**List of contents**

**Supplementary Fig. 1** Hydrocarbons conversions and product selectivity as a function of temperature over BN under "$C_2H_6$-$C_3H_6$" mode.

**Supplementary Fig. 2** $C_3H_8$ conversion under the simulated R2 inlet gas with and without added $C_3H_6$.

**Supplementary Fig. 3** $C_3H_8$ conversion in "Single-$C_3H_8$ (8 sccm)", "Single-$C_3H_8$ (10.5 sccm)" and "$C_3H_8$-$C_3H_6$" modes.

**Supplementary Fig. 4** FT-IR spectra of the fresh BN, activated BN, spent BN in the Single-$C_3H_8$ mode (Single-$C_3H_8$-BN), and spent BN in the $C_3H_8$-$C_3H_6$ mode ($C_3H_8$-$C_3H_6$-BN).

**Supplementary Fig. 5** XRD patterns of the fresh BN, activated BN, spent BN in the Single-$C_3H_8$ mode (Single-$C_3H_8$-BN), and spent BN in the $C_3H_8$-$C_3H_6$ mode ($C_3H_8$-$C_3H_6$-BN).

**Supplementary Fig. 6** $NH_3$-TPD results of the fresh BN, activated BN, spent BN in the Single-$C_3H_8$ mode (Single-$C_3H_8$-BN), and spent BN in the $C_3H_8$-$C_3H_6$ mode ($C_3H_8$-$C_3H_6$-BN).

**Supplementary Fig. 7** SEM images of the fresh BN, activated BN, spent BN in the Single-$C_3H_8$ mode (Single-$C_3H_8$-BN), and spent BN in the $C_3H_8$-$C_3H_6$ mode ($C_3H_8$-$C_3H_6$-BN).

**Supplementary Fig. 8** "Single-$C_3H_6$" temperature-programmed reaction.

**Supplementary Fig. 9** "Single-$C_2H_6$" temperature-programmed reaction.

**Supplementary Fig. 10** Dependences of alkane reaction rate on olefin reaction rate

when different $p_{alkane}/p_{olefin}$ were employed in (a) "$C_2H_6$-$C_3H_6$" mode and (b) "$C_2H_6$-$C_3H_6$" mode.

**Supplementary Fig. 11** (a) Propane conversion as a function of temperature and (b) propylene selectivity as function of propane conversion at various WHSV.

**Supplementary Fig. 12** (a) $C_3H_8$ conversion as a function of temperature at various WHSV in "$C_3H_8$- $C_3H_6$" mode. (b) $C_3H_6$, (c) $C_2H_4$, and (d) CO selectivity as function of propane conversion at various WHSV in "$C_3H_8$-$C_3H_6$" mode.

**Supplementary Fig. 13** Gas profile in transient pulses of $C_3H_6$ over BN. Signals of $C_3H_6$ (m/z = 41) and PO (m/z = 58) were measured *via* mass spectrometer.

**Supplementary Fig. 14** Gas profile in transient pulses of $C_3D_8$ with the environment for oxidation of propylene over BN at 490 °C.

**Supplementary Fig. 15** Gas profile in transient pulses of $C_3D_8$ over BN.

**Supplementary Fig. 16** Gas profile in transient pulses of $C_3D_8$ with the environment for oxidation of ethylene over BN at 520 °C.

**Supplementary Fig. 17** Products distribution for PO oxidation reaction at (a) 450 °C and (b) 510 °C.

**Supplementary Fig. 18** Propylene and propane temperature-programmed desorption over BN.

**Supplementary Fig. 19** $C_3H_8$ and $C_3H_6$ conversions as a function of temperature in the empty reactor.

**Supplementary Fig. 20** Energy profile for the $C_3H_7O$-OH bond dissociation in the singlet (black) and triplet (red) state, respectively.

**Supplementary Fig. 21** The products selectivity and propane conversion in "Single-$C_3H_8$" and "$C_3H_8$-$C_3H_6$" modes at 490 °C.

**Supplementary Fig. 22** The key stationary points and relative free energies ($\Delta G^{\neq}$ and $\Delta G$, in kcal/mol) in the abstraction of (a) primary and (b) secondary C-H bond by triplet $O_2$ to form peroxy radical in the gas phase.

**Supplementary Fig. 23** The key stationary points and relative free energies ($\Delta G^{\neq}$ and $\Delta G$, in kcal/mol) in the oxidation of propyl radical with the (a) primary and (b) secondary propyl radicals to form alkoxyl radical in the gas phase.

**Supplementary Fig. 24** Alkane conversions and product selectivity in "$C_2H_6$-$C_3H_8$" mode as a function of temperature over BN.

**Supplementary Fig. 25** XRD patterns of BN, V-$Al_2O_3$, and Li-MgO.

**Supplementary Fig. 26** (a, b) Alkanes conversions and olefins productivity in "$C_2H_6$-$C_3H_8$", "Single-$C_2H_6$" and "Single-$C_3H_8$" modes over reference catalysts.

**Supplementary Fig. 27** Dependences of ethane and propane conversion on temperature over (a) V-$Al_2O_3$ and (b) Li-MgO in different feeding modes.

**Supplementary Fig. 28** Olefins selectivity as a function of alkanes conversion in "$C_2H_6$-$C_3H_8$" mode over BN, V-$Al_2O_3$, and Li-MgO.

**Supplementary Fig. 29** $C_2H_6$ conversion in "Single-$C_2H_6$ (8 sccm)", "Single-$C_2H_6$ (10.5 sccm)" and "$C_2H_6$-$C_3H_8$" modes.

**Supplementary Fig. 30** $C_2H_6$ and $C_3H_8$ conversions as a function of temperature in the empty reactor.

**Supplementary Fig. 31** Fitting product selectivity as a function of propane conversion

over (a) BN, (c) V-$Al_2O_3$, and (e) Li-MgO. Dependences of ethylene selectivity on ethane conversion in "$C_2H_6$-$C_3H_8$" and "Single-$C_2H_6$" modes over (b) BN, (d) V-$Al_2O_3$, and (f) Li-MgO.

**Supplementary Fig. 32** Dependences of alkanes conversions on temperature over BN in "$CH_4$-$C_2H_6$", "Single-$CH_4$", and "Single-$C_2H_6$" modes.

**Supplementary Table 1** Conversion of reactants under different "alkane-olefin" atmosphere.

**Supplementary Table 2** Specific treatments of catalysts.

**Supplementary Table 3** Alkane conversions, products distribution, and space-time yield at similar alkane conversions under different feeding modes.

**Supplementary References**

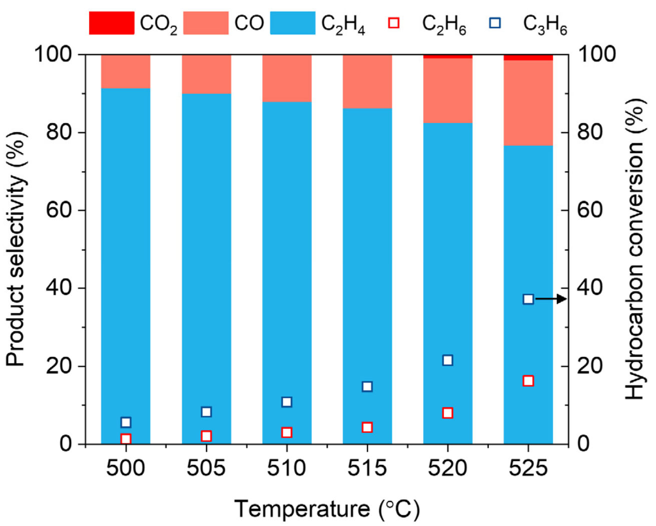


**Supplementary Fig. 1** Hydrocarbons conversions and product selectivity as a function of temperature over BN under "$C_2H_6$-$C_3H_6$" mode. $F_{total}$ = 40 mL min$^{-1}$, $C_2H_6$: $C_3H_6$: $O_2$: $N_2$ = 8: 2.5: 8: 21.5.

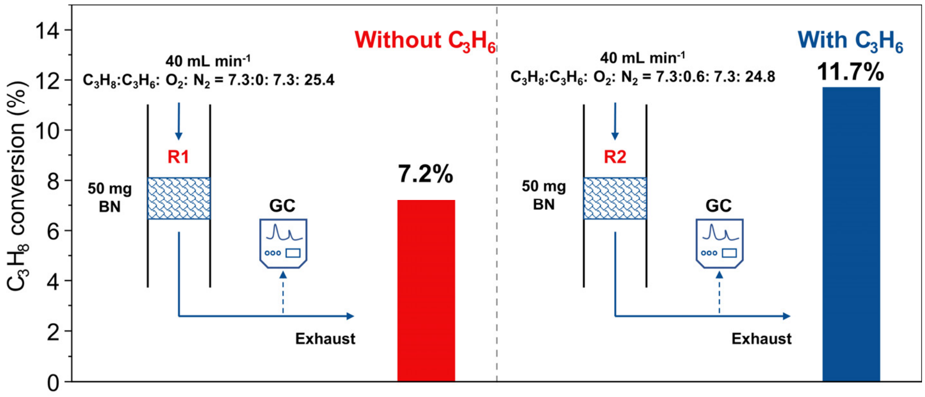


**Supplementary Fig. 2** $C_3H_8$ conversion under the simulated R2 inlet gas with and without added $C_3H_6$.

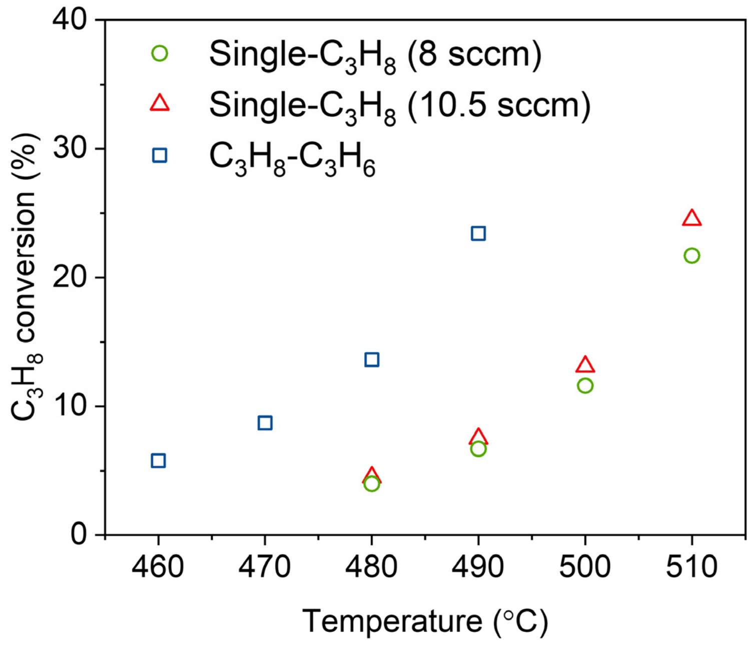


**Supplementary Fig. 3** $C_3H_8$ conversion in "Single-$C_3H_8$ (8 sccm)", "Single-$C_3H_8$ (10.5 sccm)" and "$C_3H_8$-$C_3H_6$" modes. $F_{total}$ = 40 mL $min^{-1}$. "Single-$C_3H_8$ (8 sccm)", $C_3H_8$: $O_2$: $N_2$ = 8: 8: 24; "Single-$C_3H_8$ (10.5 sccm)", $C_3H_8$: $O_2$: $N_2$ = 10.5: 8: 21.5; "$C_3H_8$-$C_3H_6$", $C_3H_8$: $C_3H_6$: $O_2$: $N_2$ = 8: 2.5: 8: 21.5.

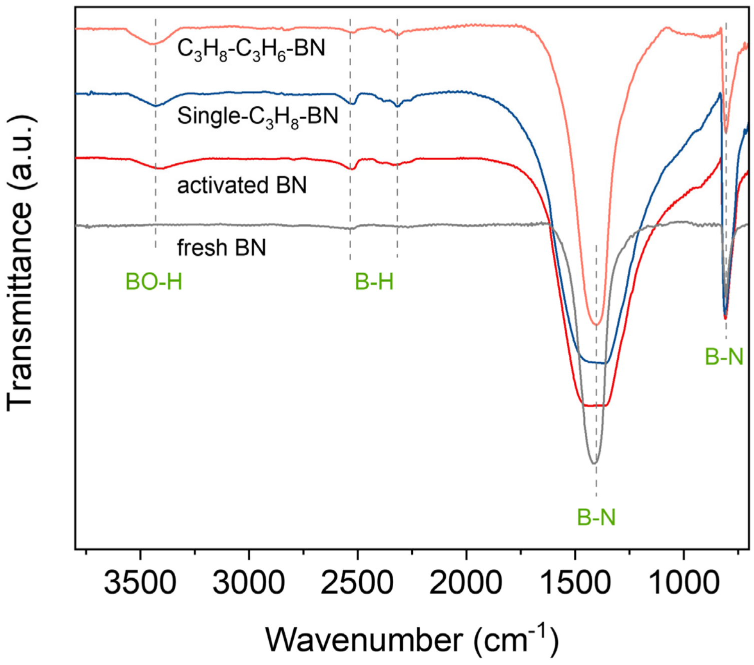


**Supplementary Fig. 4** FT-IR spectra of the fresh BN, activated BN, spent BN in the Single-$C_3H_8$ mode (Single-$C_3H_8$-BN), and spent BN in the $C_3H_8$-$C_3H_6$ mode ($C_3H_8$-$C_3H_6$-BN).

**Note:** The fresh BN exhibited the characteristic in-plane B-N transverse stretching vibration at ~1390 $cm^{-1}$ and out-of-plane bending vibration at ~806 $cm^{-1}$, respectively.[1] The spectra of activated BN exhibited new peaks belonging to BO-H vibration at ~3400 $cm^{-1}$ and B-H vibration at ~2520 $cm^{-1}$ and ~2320 $cm^{-1}$, illustrating boron sites were hydroxylated and hydrogenated during the ODH reaction.[2,3] Single-$C_3H_8$-BN and $C_3H_8$-$C_3H_6$-BN showed no difference compared with activated BN.

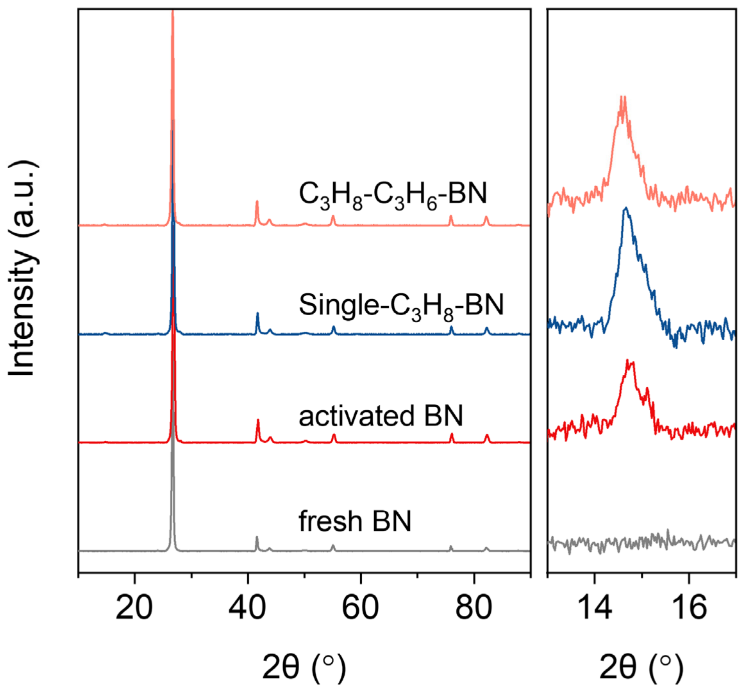


**Supplementary Fig. 5** XRD patterns of the fresh BN, activated BN, spent BN in the Single-$C_3H_8$ mode (Single-$C_3H_8$-BN), and spent BN in the $C_3H_8$-$C_3H_6$ mode ($C_3H_8$-$C_3H_6$-BN).

**Note:** The diffraction peaks at 2θ = 26.8°, 41.5°, and 43.9° were observed in fresh BN, corresponding to the (002), (100), and (101) crystal plane of h-BN. After activation, the diffraction peaks assigned to h-BN were retained, indicating the structure of the bulk phase was not destroyed during the ODH reaction. A new diffraction peak at 2θ = 14.6° appeared in the activated BN pattern, which was assigned to $BO_x$.[4] Single-$C_3H_8$-BN and $C_3H_8$-$C_3H_6$-BN showed no difference compared with activated BN.

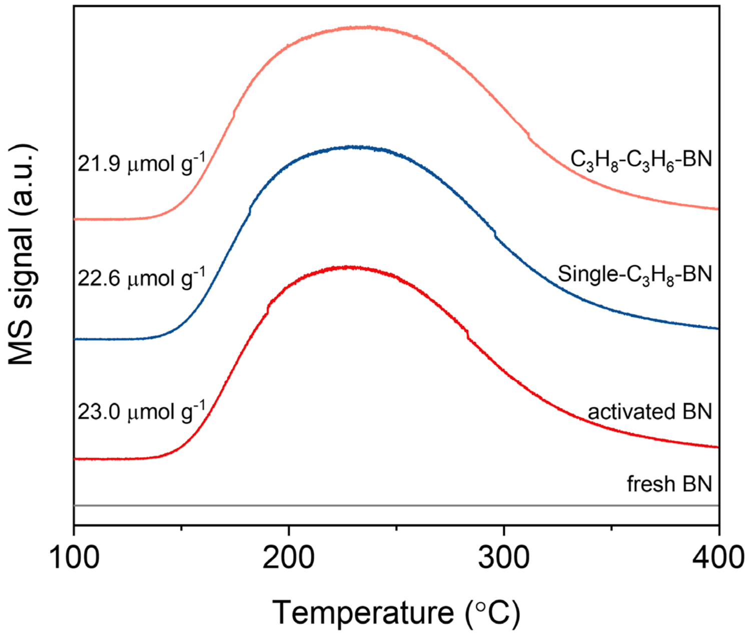


**Supplementary Fig. 6** $NH_3$-TPD results of the fresh BN, activated BN, spent BN in the Single-$C_3H_8$ mode (Single-$C_3H_8$-BN), and spent BN in the $C_3H_8$-$C_3H_6$ mode ($C_3H_8$-$C_3H_6$-BN).

**Note:** The profiles of $NH_3$ desorption during TPD indicated that the fresh BN had no acidity and one acid site was generated in activated BN. The desorption temperature of the acid site was about 230 °C and the $NH_3$ desorption capacity was 23.0 μmol $g^{-1}$. When the activated BN were treated for another 3 h under the two atmospheres ("Single-$C_3H_8$", "$C_3H_8$-$C_3H_6$"), the desorption temperature was about 230 °C and the $NH_3$ desorption capacity was almost unchanged.

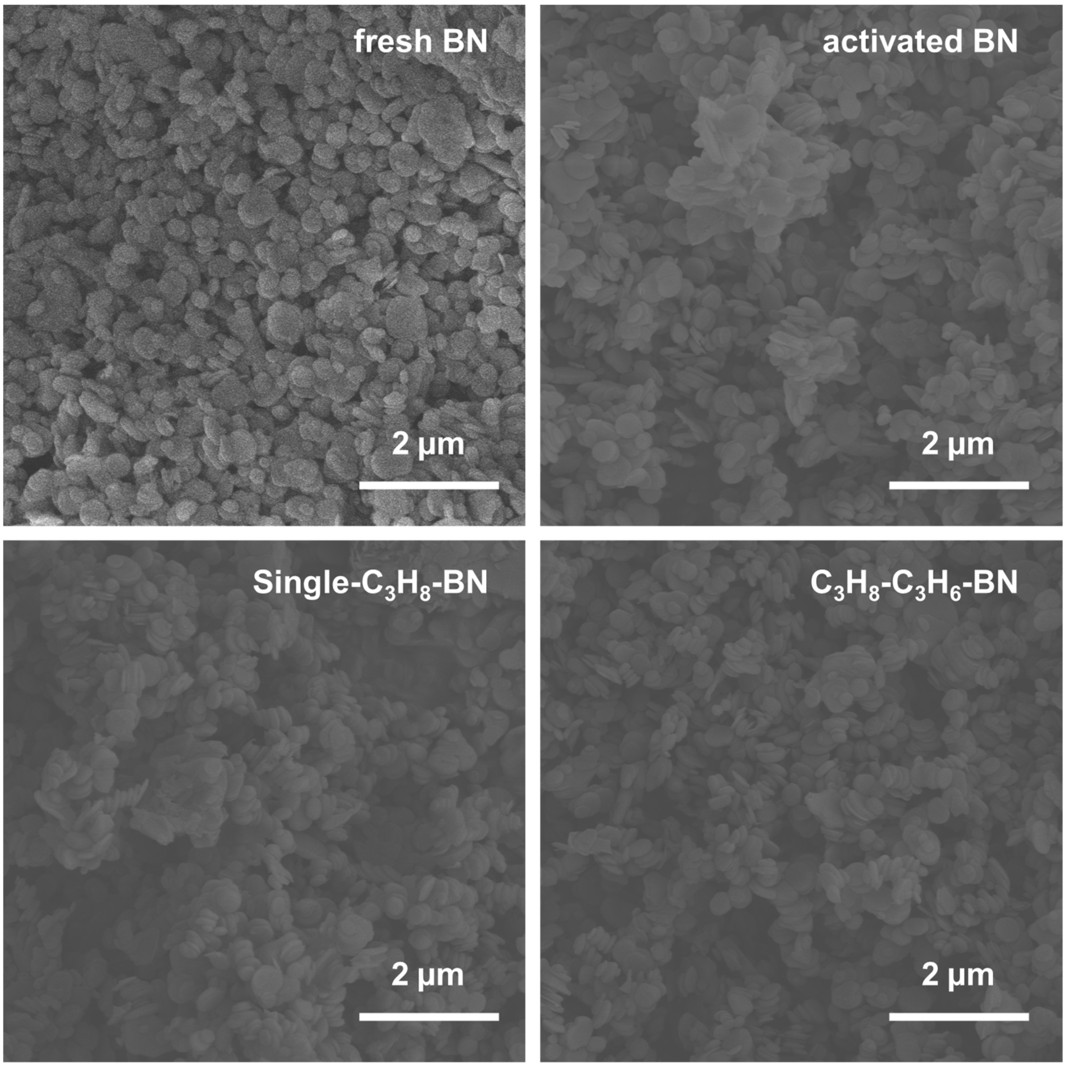


**Supplementary Fig. 7** SEM images of the fresh BN, activated BN, spent BN in the Single-$C_3H_8$ mode (Single-$C_3H_8$-BN), and spent BN in the $C_3H_8$-$C_3H_6$ mode ($C_3H_8$-$C_3H_6$-BN).

**Note:** The fresh BN showed that the boron nitride sheets were uniformly dispersed. After the activation in the ODH reaction environment, the sheets aggregated into larger bulks, indicating that some cohesive $BO_x$ species were formed. When the activated BN were treated for another 3 h under the two atmospheres ("Single-$C_3H_8$", "$C_3H_8$-$C_3H_6$"), the morphology was almost unchanged.

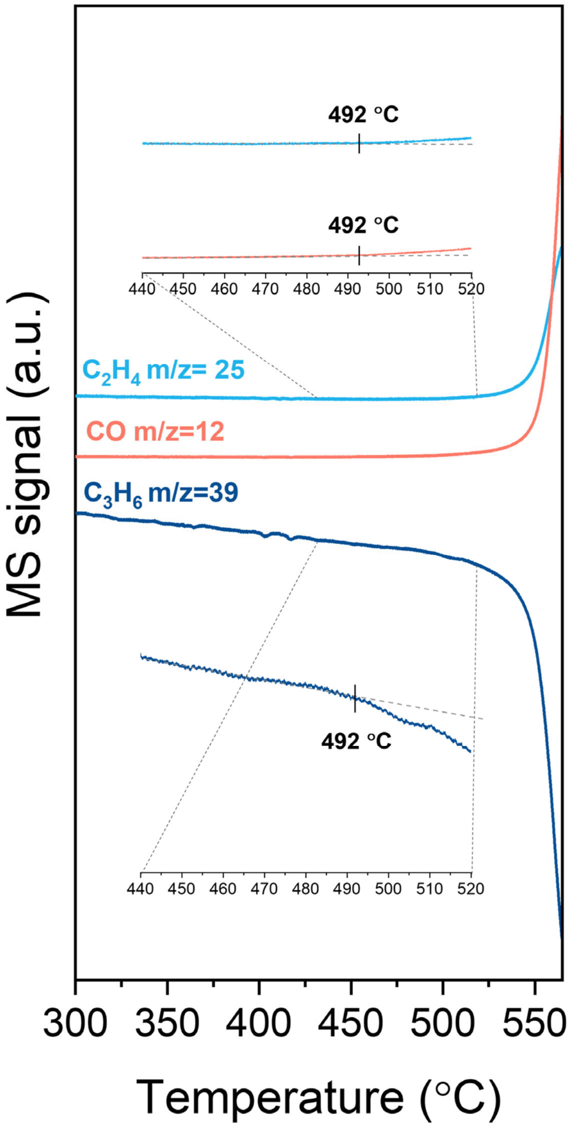


**Supplementary Fig. 8** "Single-$C_3H_6$" temperature-programmed reaction. Signals of $C_2H_4$ (m/z = 25), CO (m/z = 12), and $C_3H_6$ (m/z = 39) were measured *via* mass spectrometer. $F_{total}$ = 40 mL min$^{-1}$, $C_3H_6$: $O_2$: $N_2$ = 2.5: 8: 29.5, $m_{cat}$ = 100 mg.

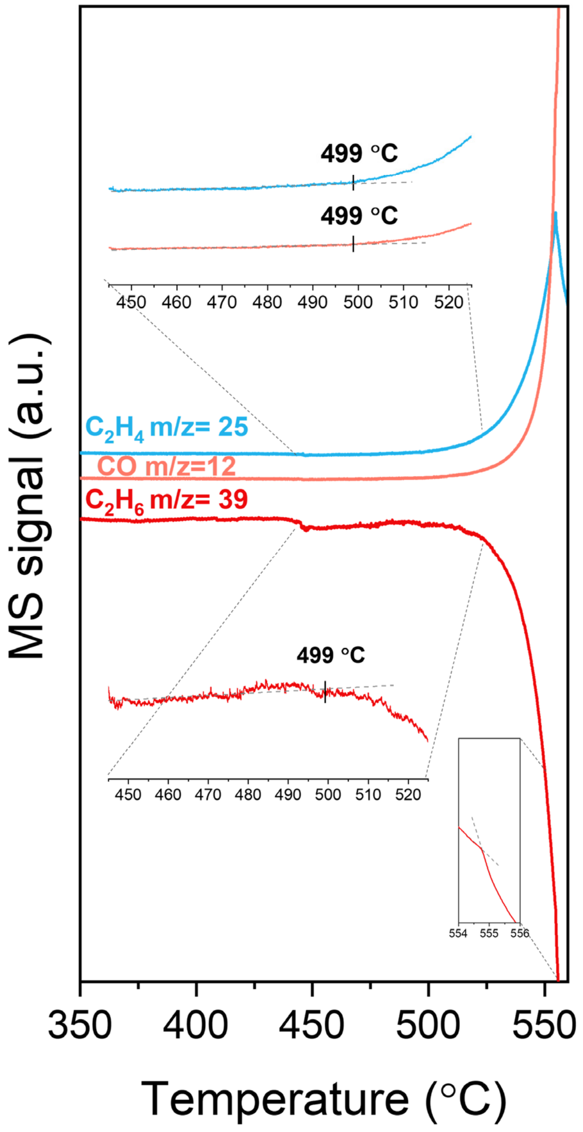


**Supplementary Fig. 9** "Single-$C_2H_6$" temperature-programmed reaction. Signals of $C_2H_4$ (m/z = 25), CO (m/z = 12), and $C_2H_6$ (m/z = 30) were measured *via* mass spectrometer. $F_{total}$ = 40 mL $min^{-1}$, $C_2H_6$: $O_2$: $N_2$ = 8: 8: 24., $m_{cat}$ = 100 mg.

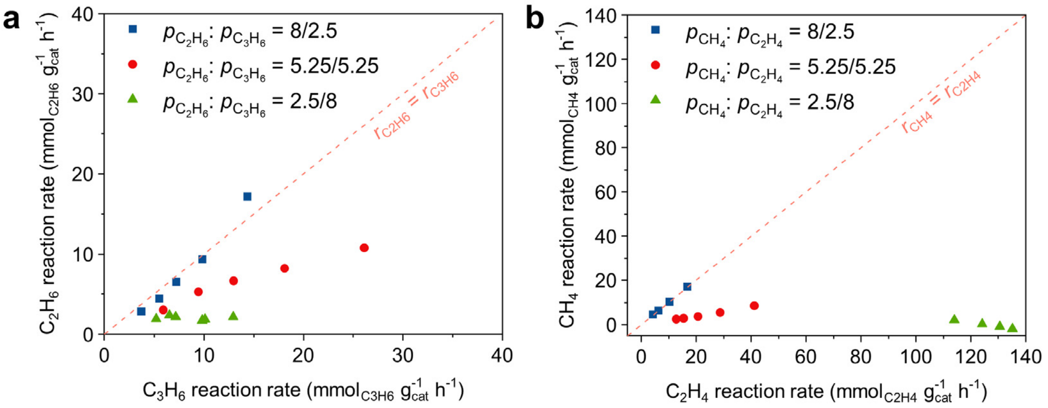


**Supplementary Fig. 10** Dependences of alkane reaction rate on olefin reaction rate when different $p_{alkane}/p_{olefin}$ were employed in (a) "$C_2H_6$-$C_3H_6$" mode and (b) "$C_2H_6$-$C_3H_6$" mode. $F_{total}$ = 40 mL min$^{-1}$, $O_2$: $N_2$ = 8: 21.5, $m_{cat}$ = 100 mg.

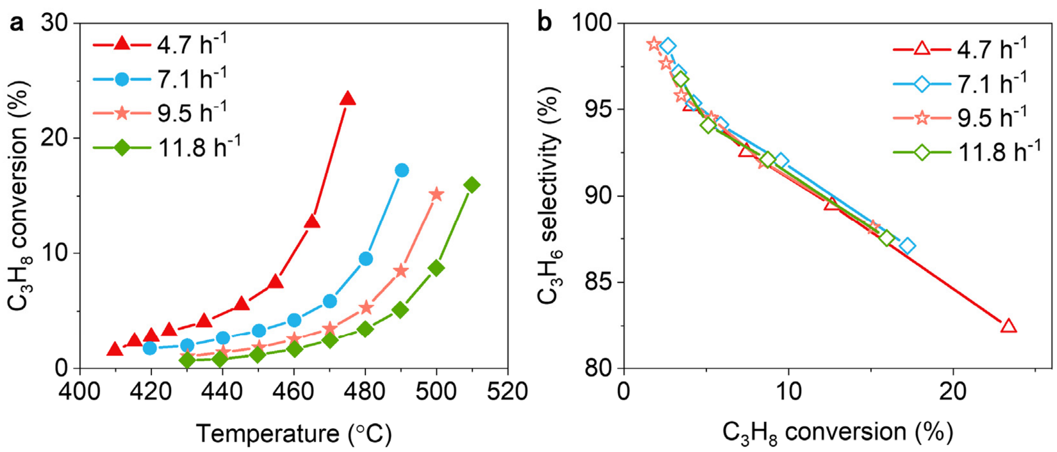


**Supplementary Fig. 11** (a) $C_3H_8$ conversion as a function of temperature and (b) $C_3H_6$ selectivity as function of propane conversion at various WHSV in "Single-$C_3H_8$" mode. $C_3H_8$: $O_2$: $N_2$ = 1: 1: 3; reaction temperature: 410-510 °C.

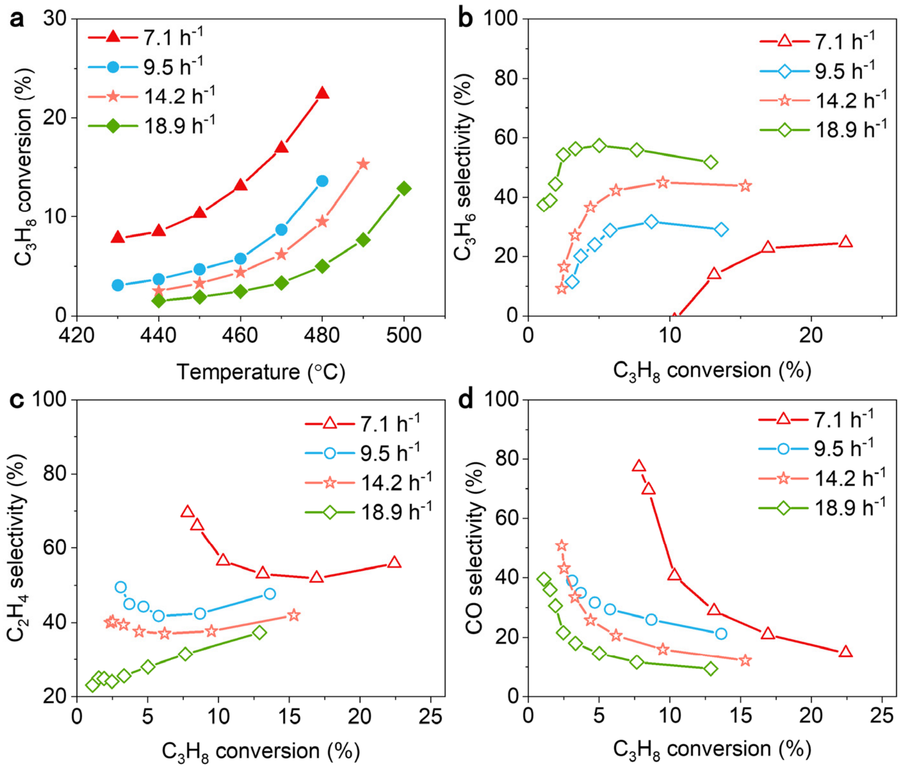


**Supplementary Fig. 12** (a) $C_3H_8$ conversion as a function of temperature at various WHSV in "$C_3H_8$- $C_3H_6$" mode. (b) $C_3H_6$, (c) $C_2H_4$, and (d) CO selectivity as function of propane conversion at various WHSV in "$C_3H_8$- $C_3H_6$" mode. $C_3H_8$: $C_3H_6$: $O_2$: $N_2$ = 8: 2.5: 8: 21.5; reaction temperature: 430-500 °C. Because we could not know the consumption of co-fed propylene and the production of propylene formed from propane in the "$C_3H_8$-$C_3H_6$" mode, the product distribution could only be obtained by calculating the net production of $C_3H_6$, where the conversion of co-fed propylene was assumed to be 0. Note that the method would underestimate propylene selectivity because it didn't consider the propylene that was consumed to promote more conversion of propane before diffusing from the reactor. Furthermore, ethylene and $CO_x$ generated from co-fed propylene were taken into account in the calculation of product distribution, leading to an overestimation of their selectivity.

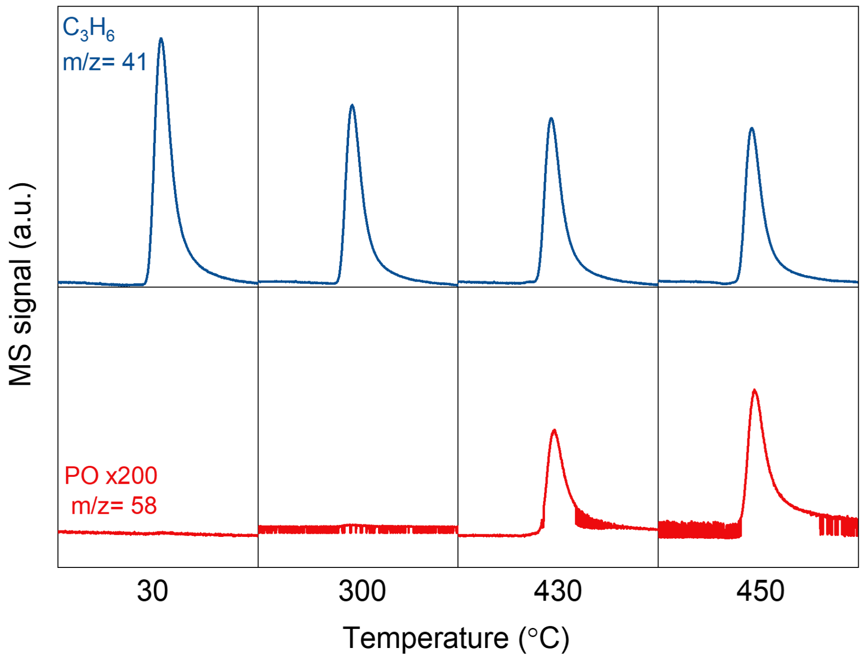


**Supplementary Fig. 13** Gas profile in transient pulses of $C_3H_6$ over BN. Signals of $C_3H_6$ (m/z = 41) and PO (m/z = 58) were measured *via* mass spectrometer. $F_{total}$ = 40 mL $min^{-1}$, $C_3H_8$: $O_2$: $N_2$ = 8: 8: 24; $m_{BN}$ = 100 mg; pulse value: 1 mL each time.

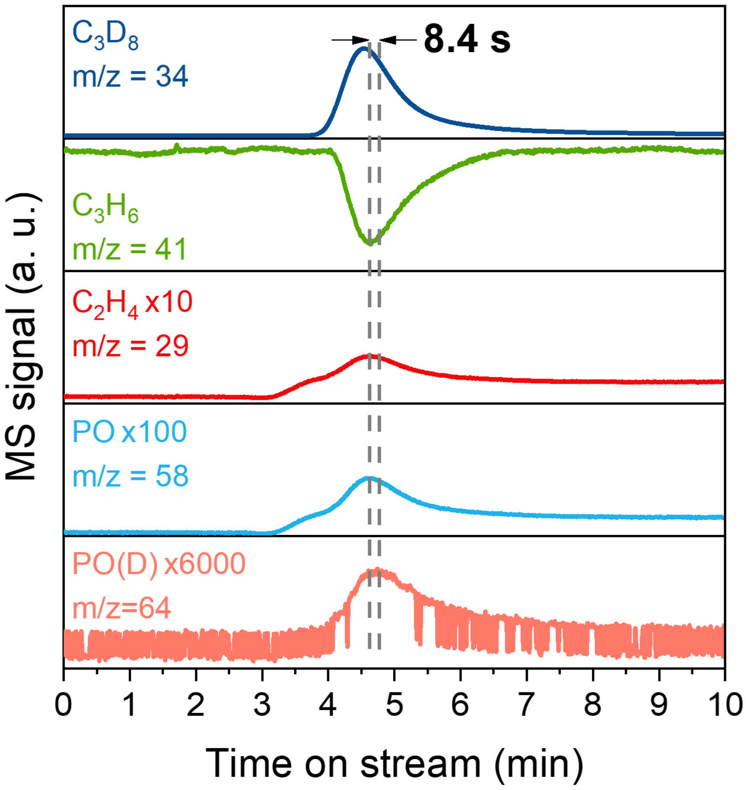


**Supplementary Fig. 14** Gas profile in transient pulses of $C_3D_8$ with the environment for oxidation of propylene over BN at 490 °C. $F_{total}$ = 40 mL min$^{-1}$, $C_3H_6$: $O_2$: $N_2$ = 2.5: 8: 29.5; $m_{cat}$ = 100 mg; pulse value: 1 mL each time.

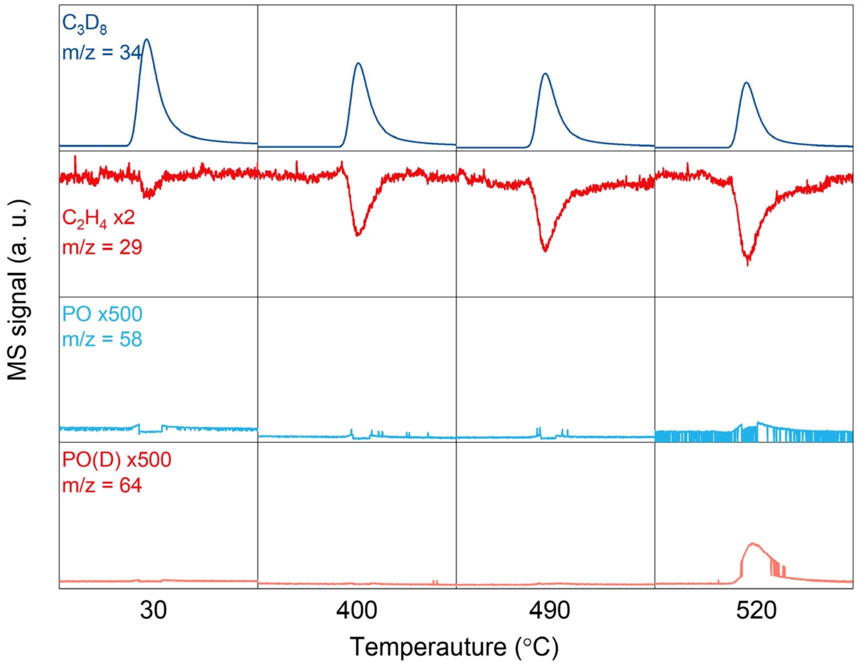


**Supplementary Fig. 15** Gas profile in transient pulses of $C_3D_8$ over BN. Signals of $C_3D_8$ (m/z = 34), $C_2H_4$ (m/z = 29), PO (m/z = 58), and PO(D) (m/z = 64) were measured *via* mass spectrometer. $F_{total}$ = 40 mL min$^{-1}$, $C_2H_4$: $O_2$: $N_2$ = 2.5: 8: 29.5; $m_{cat}$ = 100 mg; pulse value: 1 mL each time.

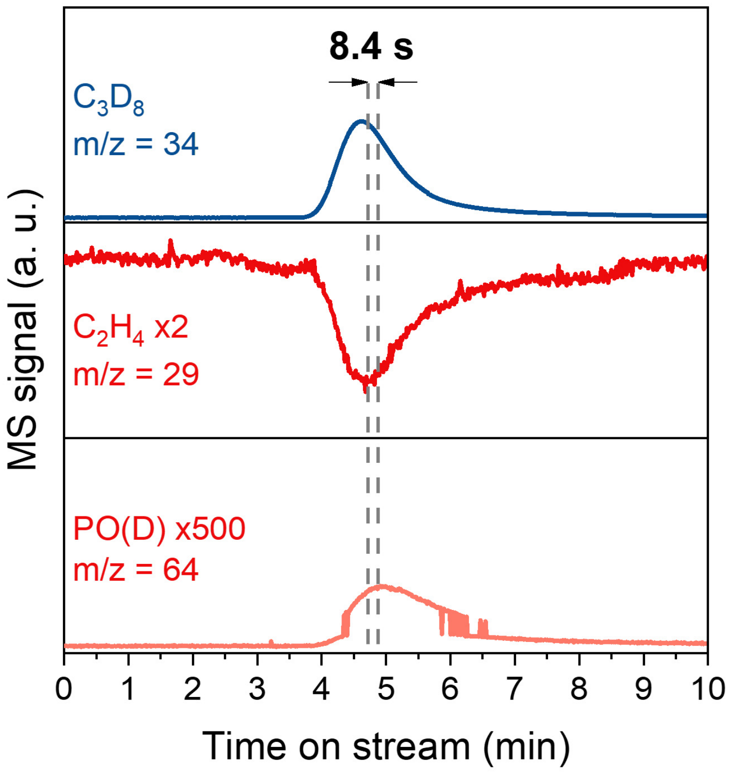


**Supplementary Fig. 16** Gas profile in transient pulses of $C_3D_8$ with the environment for oxidation of ethylene over BN at 520 °C. $F_{total}$ = 40 mL min$^{-1}$, $C_2H_4$: $O_2$: $N_2$ = 2.5: 8: 29.5; $m_{cat}$ = 100 mg; pulse value: 1 mL each time.

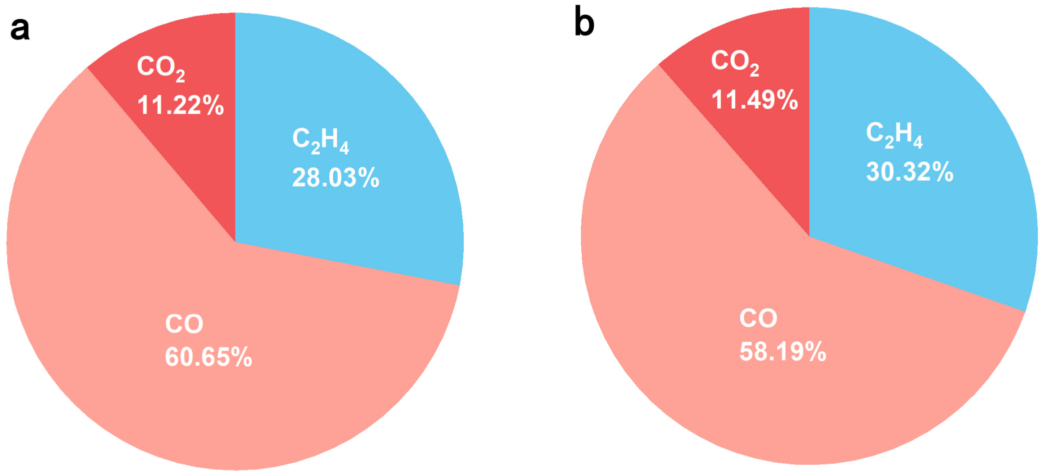


**Supplementary Fig. 17** Products distribution for PO oxidation reaction at (a) 450 °C and (b) 510 °C. $F_{total}$ = 40 mL min$^{-1}$, $O_2$: $N_2$ = 1: 4; $m_{cat}$ = 100 mg; PO was bubbled into the reactor at 0 °C.

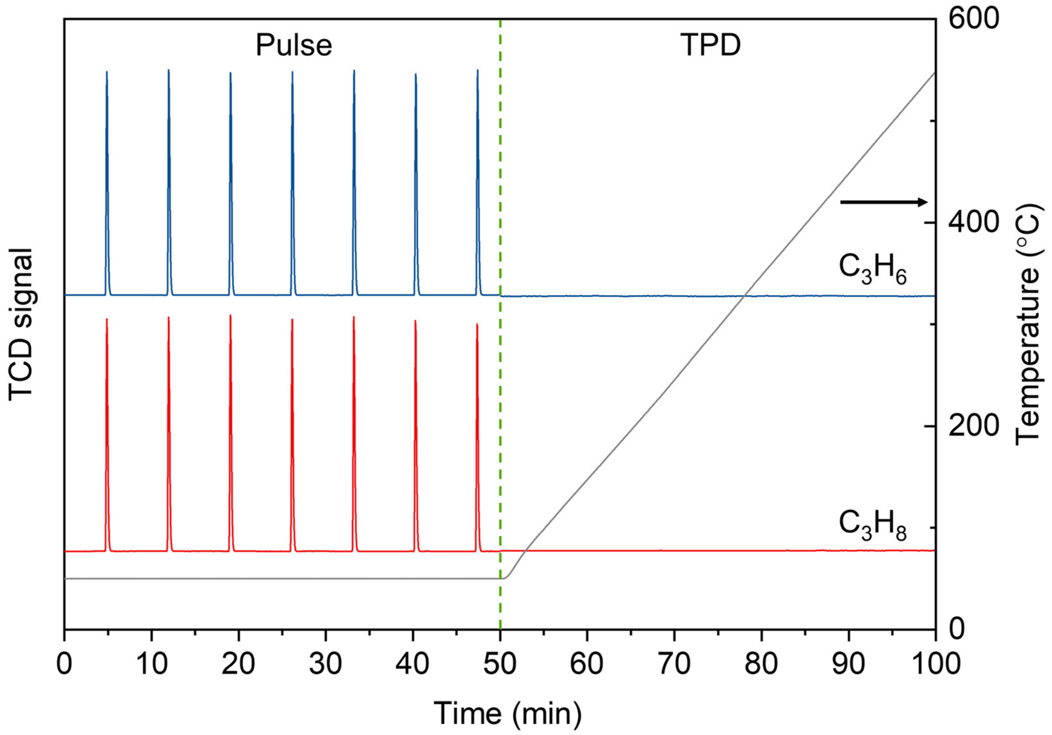


**Supplementary Fig. 18** Propylene and propane temperature-programmed desorption over BN.

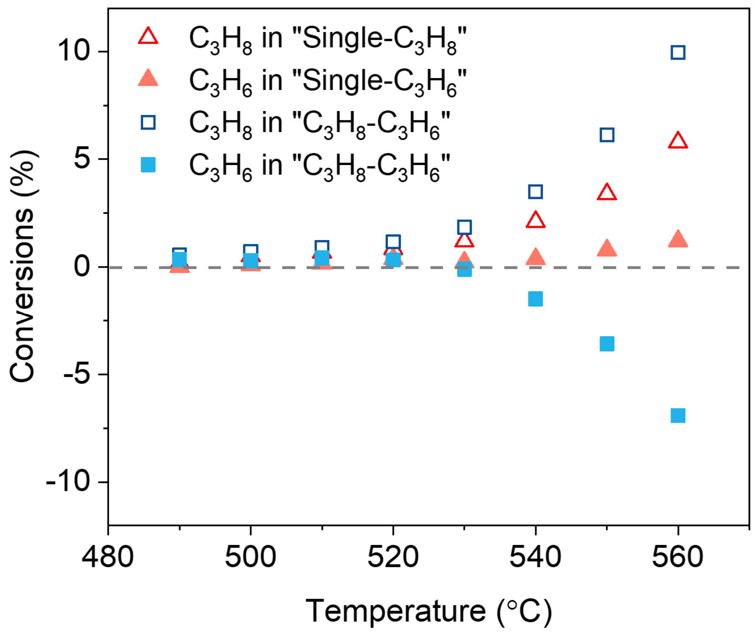


**Supplementary Fig. 19** $C_3H_8$ and $C_3H_6$ conversions as a function of temperature in the empty reactor. $F_{total}$ = 40 mL min$^{-1}$, $C_3H_8$: $C_3H_6$: $O_2$: $N_2$ = 8: 2.5: 8: 21.5.

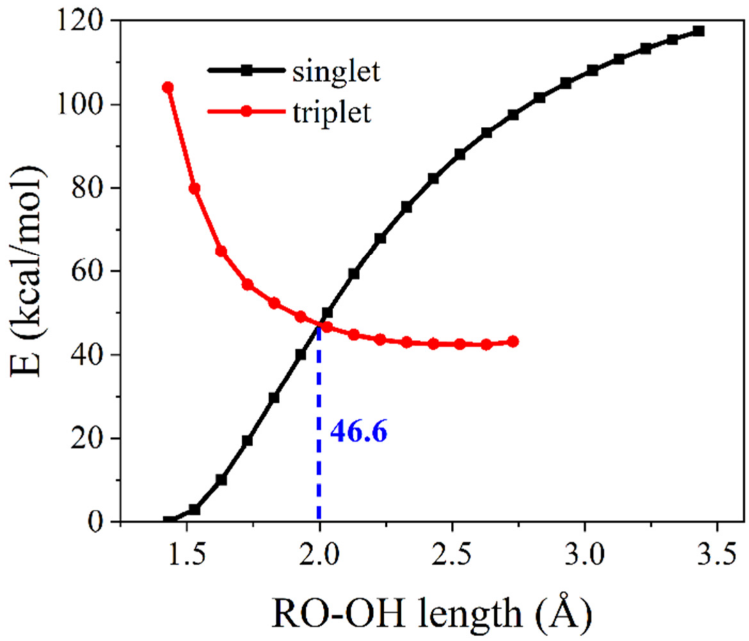


**Supplementary Fig. 20** Energy profile for the $C_3H_7O$-OH bond dissociation in the singlet (black) and triplet (red) state, respectively.

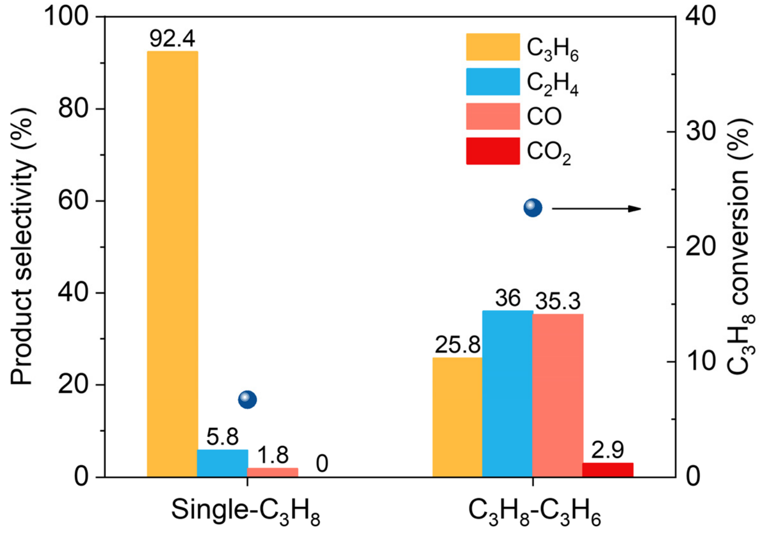


**Supplementary Fig. 21** The products selectivity and propane conversion in "Single-$C_3H_8$" and "$C_3H_8$-$C_3H_6$" modes at 490 °C. $F_{total}$ = 40 mL $min^{-1}$, "Single-$C_3H_8$", $C_3H_8$: $O_2$: $N_2$ = 8: 8: 24; "$C_3H_8$-$C_3H_6$", $C_3H_8$: $C_3H_6$: $O_2$: $N_2$ = 8: 2.5: 8: 21.5.

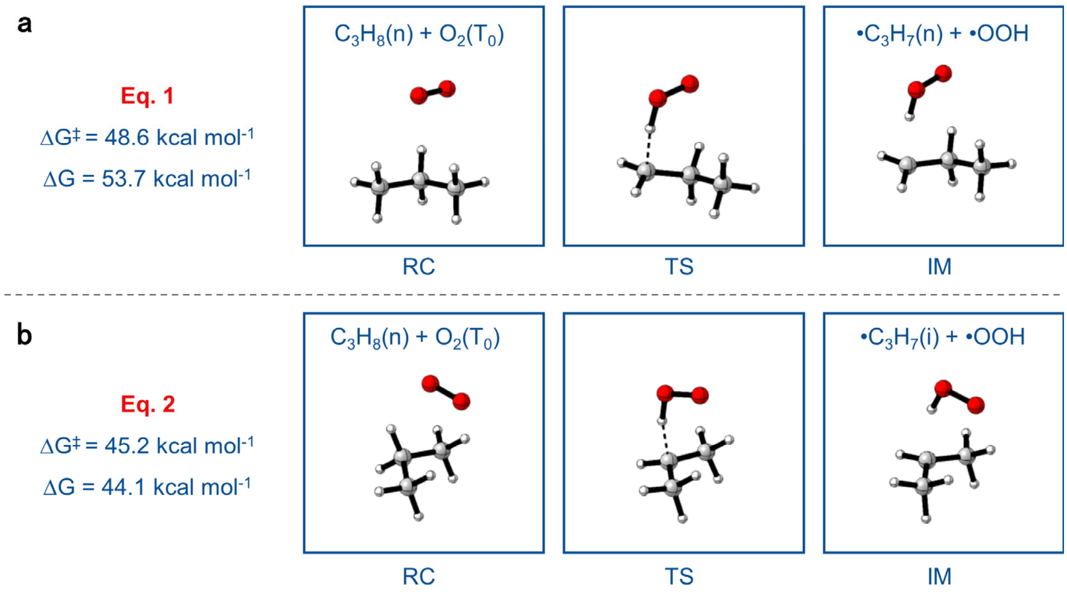


**Supplementary Fig. 22** The key stationary points and relative free energies ($\Delta G^{\neq}$ and $\Delta G$, in kcal/mol) in the abstraction of (a) primary and (b) secondary C-H bond by triplet $O_2$ to form peroxy radical in the gas phase.

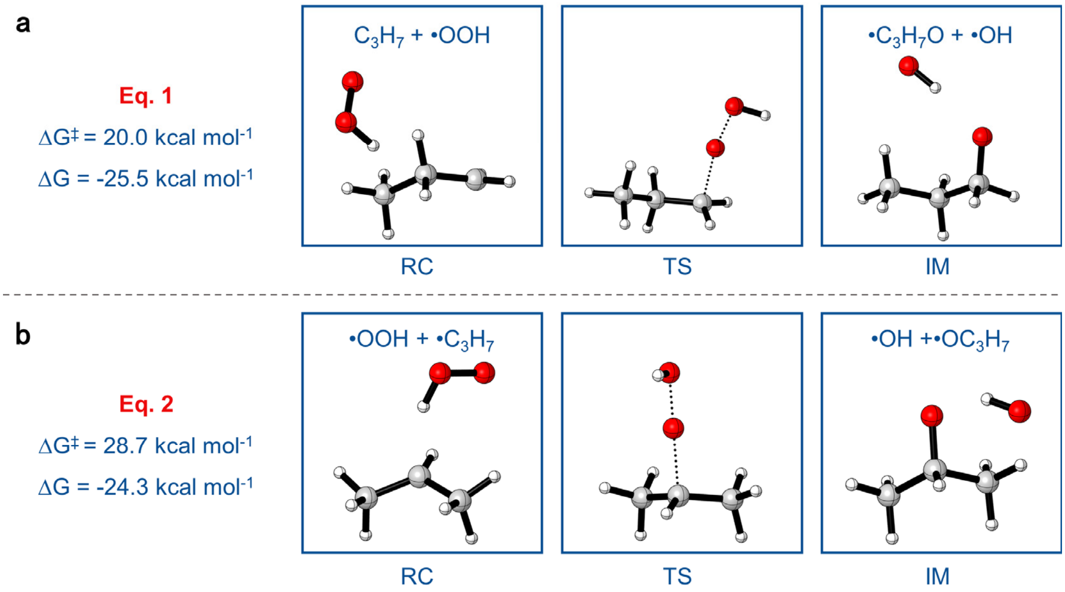


**Supplementary Fig. 23** The key stationary points and relative free energies ($\Delta G^{\neq}$ and $\Delta G$, in kcal/mol) in the oxidation of propyl radical with the (a) primary and (b) secondary propyl radicals to form alkoxyl radical in the gas phase.

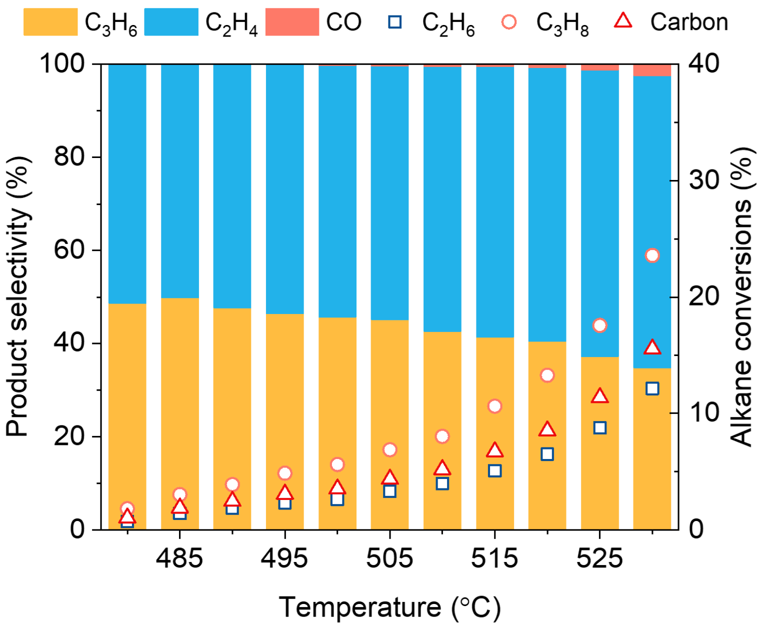


**Supplementary Fig. 24** Alkane conversions and product selectivity in "$C_2H_6$-$C_3H_8$" mode as a function of temperature over BN. $F_{total}$ = 40 mL min$^{-1}$, $C_2H_6$: $C_3H_8$: $O_2$: $N_2$ = 8: 2.5: 8: 21.5.

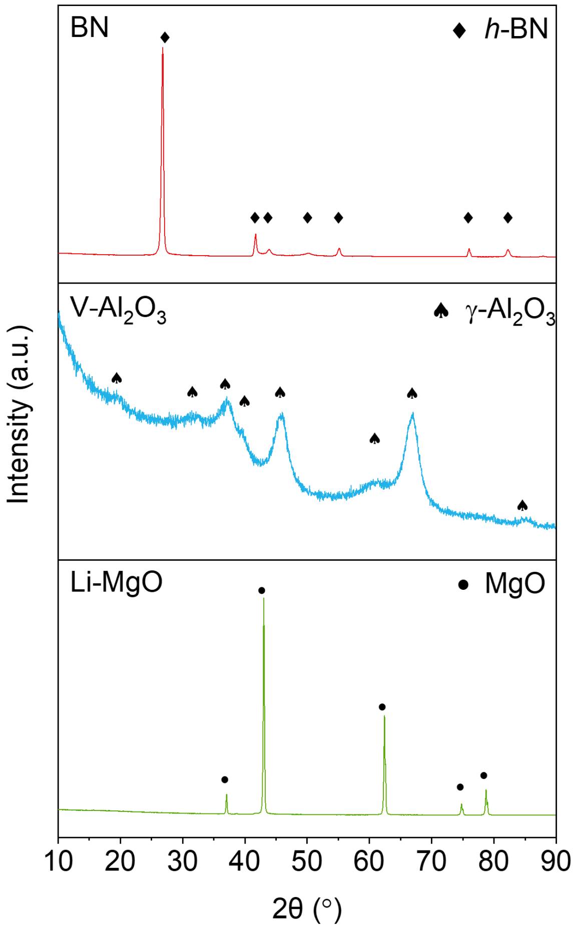


**Supplementary Fig. 25** XRD patterns of BN, V-$Al_2O_3$, and Li-MgO.

**Note:** BN showed the typical hexagonal layered structure (JCPDS 01-073-2095). For V-$Al_2O_3$, all the detected peaks were attributed to the γ-$Al_2O_3$ (JCPDS 96-120-0016), indicating that the catalysts are mainly covered by two-dimensional $VO_x$ species.[5] For Li-MgO, only diffraction peaks assigned to MgO (JCPDS 96-900-6806) were observed, demonstrating Li species were highly dispersed over the catalyst.

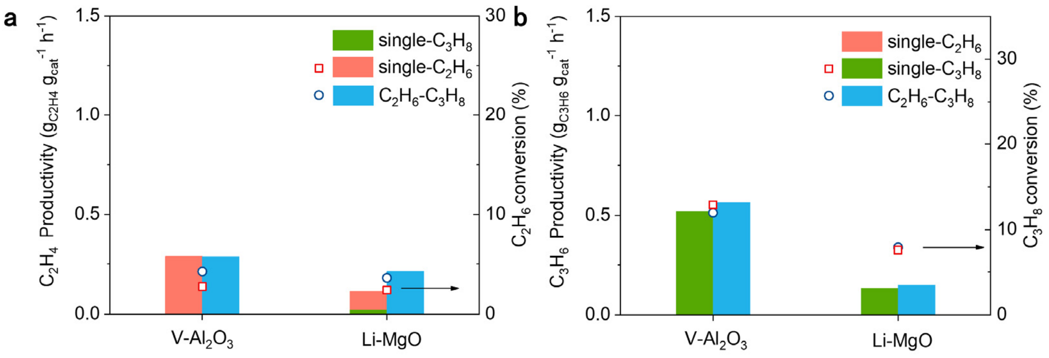


**Supplementary Fig. 26** (a, b) Alkanes conversions and olefins productivity in "$C_2H_6$-$C_3H_8$", "Single-$C_2H_6$" and "Single-$C_3H_8$" modes over reference catalysts. The reaction temperatures were 434 °C (V-$Al_2O_3$) and 565 °C (Li-MgO), respectively. $F_{total}$ = 40 mL $min^{-1}$, "Single-$C_2H_6$", $C_2H_6$: $O_2$: $N_2$ = 8: 8: 24; "Single-$C_3H_8$", $C_3H_8$: $O_2$: $N_2$ = 2.5: 8: 29.5; "$C_2H_6$-$C_3H_8$", $C_2H_6$: $C_3H_8$: $O_2$: $N_2$ = 8: 2.5: 8: 21.5.

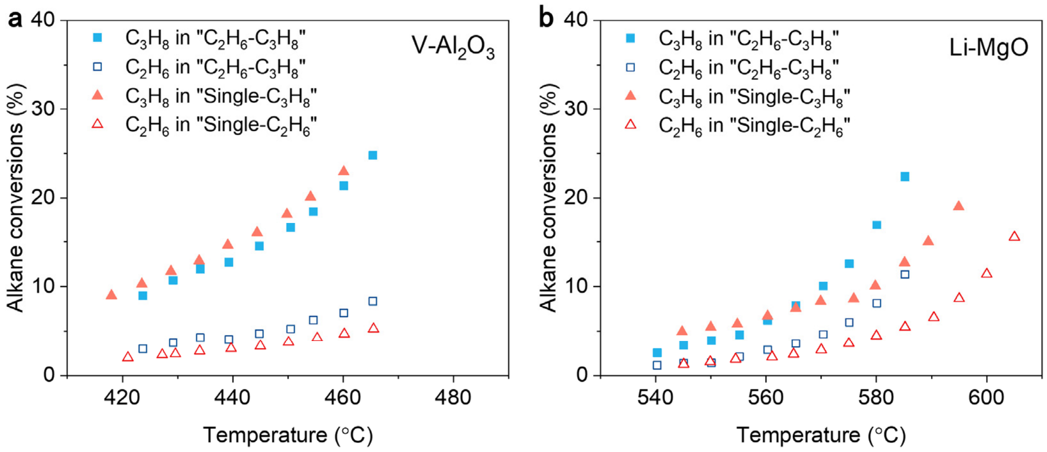


**Supplementary Fig. 27** Dependences of ethane and propane conversion on temperature over (a) V-Al$_2$O$_3$ and (b) Li-MgO in different feeding modes. $F_{total}$ = 40 mL min$^{-1}$, "Single-C$_2$H$_6$", $C_2H_6$: $O_2$: $N_2$ = 8: 8: 24; "Single-C$_3$H$_8$", $C_3H_8$: $O_2$: $N_2$ = 2.5: 8: 29.5; "C$_2$H$_6$-C$_3$H$_8$", $C_2H_6$: $C_3H_8$: $O_2$: $N_2$ = 8: 2.5: 8: 21.5.

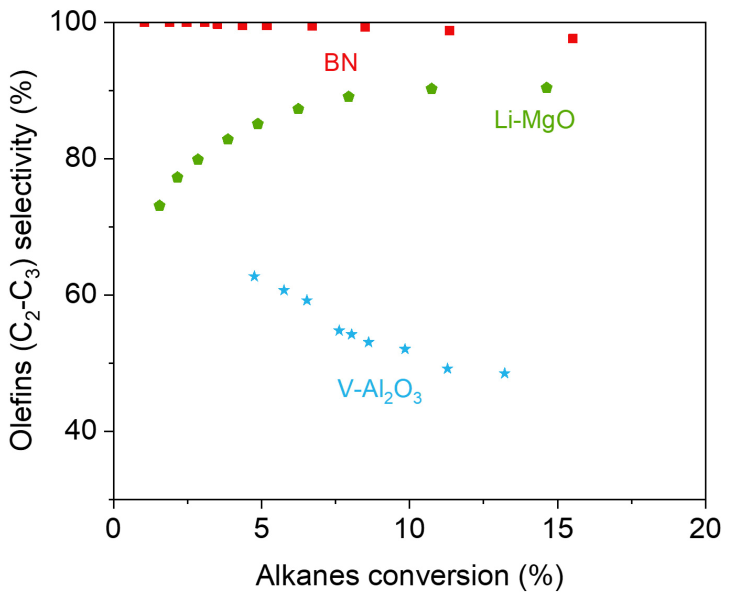


**Supplementary Fig. 28** Olefins selectivity as a function of alkanes conversion in "$C_2H_6$-$C_3H_8$" mode over BN, V-$Al_2O_3$, and Li-MgO. $F_{total}$ = 40 mL min$^{-1}$, $C_2H_6$: $C_3H_8$: $O_2$: $N_2$ = 8: 2.5: 8: 21.5.

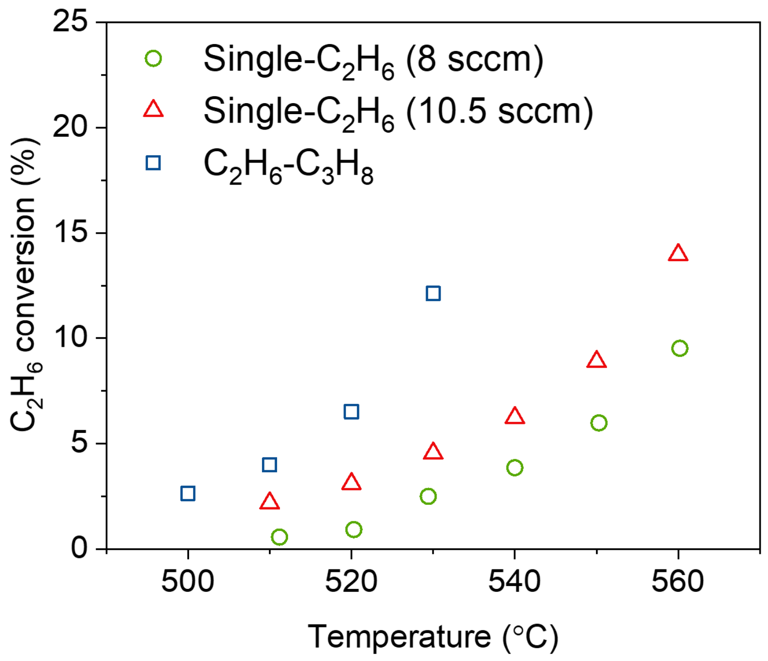


**Supplementary Fig. 29** $C_2H_6$ conversion in "Single-$C_2H_6$ (8 sccm)", "Single-$C_2H_6$ (10.5 sccm)" and "$C_2H_6$-$C_3H_8$" modes. $F_{total}$ = 40 mL min$^{-1}$. "Single-$C_2H_6$ (8 sccm)", $C_2H_6$: $O_2$: $N_2$ = 8: 8: 24; "Single-$C_2H_6$ (10.5 sccm)", $C_2H_6$: $O_2$: $N_2$ = 10.5: 8: 21.5; "$C_2H_6$-$C_3H_8$", $C_2H_6$: $C_3H_8$: $O_2$: $N_2$ = 8: 2.5: 8: 21.5.

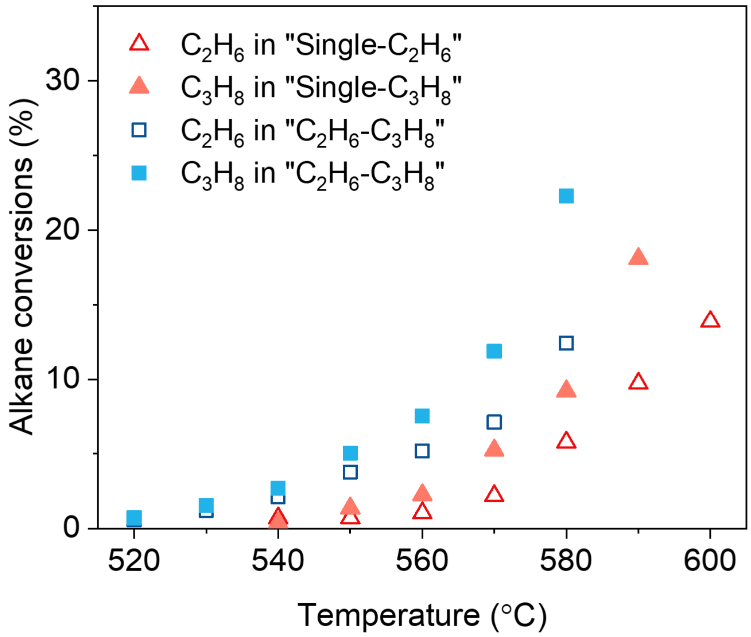


**Supplementary Fig. 30** $C_2H_6$ and $C_3H_8$ conversions as a function of temperature in the empty reactor. $F_{total}$ = 40 mL min$^{-1}$, $C_2H_6$: $C_3H_8$: $O_2$: $N_2$ = 8: 2.5: 8: 21.5.

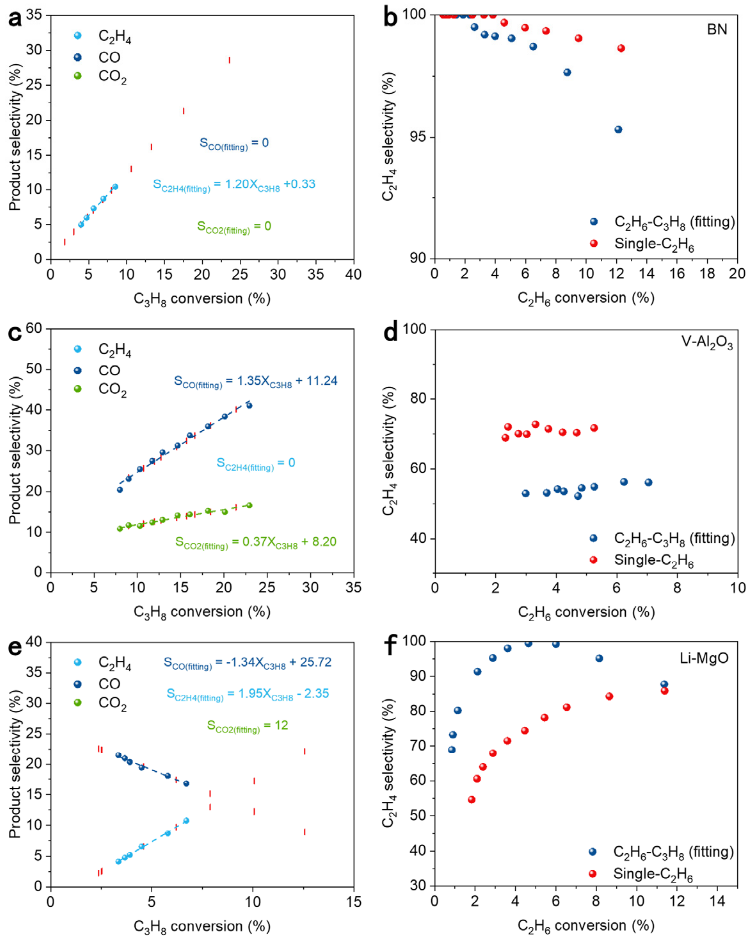


**Supplementary Fig. 31** Fitting product selectivity as a function of propane conversion over (a) BN, (c) V-$Al_2O_3$, and (e) Li-MgO. Dependences of ethylene selectivity on ethane conversion in "$C_2H_6$-$C_3H_8$" and "Single-$C_2H_6$" modes over (b) BN, (d) V-$Al_2O_3$, and (f) Li-MgO.

**Note:** Independent products selectivity was unable directly obtained because ethylene, carbonic oxide, and carbon dioxide could generate from both propane and ethane. Assuming that the relationship between selectivity of products from propane and propane conversion in "$C_2H_6$-$C_3H_8$" was the same as that in "Single-$C_3H_8$", the relation between selectivity of ethane-derived ethylene and the conversion of ethane in "$C_2H_6$-$C_3H_8$" was investigated. The calculation method was as follows: (1) The relationship between products selectivity and propane conversion in "Single-$C_3H_8$" was fitted into

a linear formula. (2) The propane conversion in "$C_2H_6$-$C_3H_8$" was substituted into the formula to calculate the selectivity of propane-derived products, and the productivity was further calculated. (3) The productivity of ethane-derived products was obtained by subtracting the productivity of propane-derived products from the total productivity in "$C_2H_6$-$C_3H_8$", and the selectivity of ethane-derived ethylene was further calculated.

For V-$Al_2O_3$, ethylene selectivity was reduced when adding propane whereas it was still a constant, suggesting that route of ethylene formation may be unchanged. For Li-MgO, the selectivity of ethylene showed a volcano-type dependence on the conversion of ethane in "$C_2H_6$-$C_3H_8$". However, the selectivity increased and then remained unchanged with the increase of ethane conversion in "Single-$C_2H_6$".

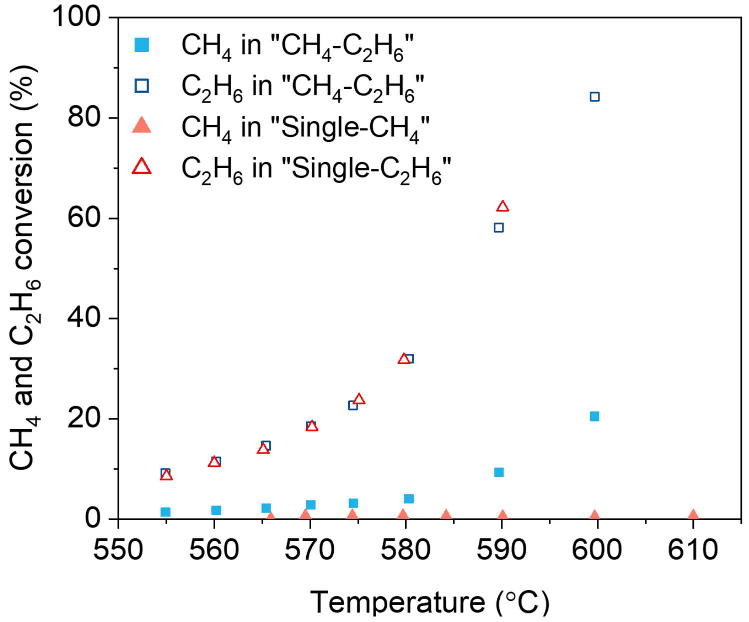


**Supplementary Fig. 32** Dependences of alkanes conversions on temperature over BN in “$CH_4$-$C_2H_6$”, “Single-$CH_4$”, and “Single-$C_2H_6$” modes. $F_{total}$ = 40 mL $min^{-1}$, “Single-$CH_4$”, $CH_4$: $O_2$: $N_2$ = 8: 8: 24; “Single-$C_2H_6$”, $C_2H_6$: $O_2$: $N_2$ = 2.5: 8: 29.5; “$CH_4$-$C_2H_6$”, $CH_4$: $C_2H_6$: $O_2$: $N_2$ = 8: 2.5: 8: 21.5.

**Supplementary Table 1** Conversion of reactants under different "alkane-olefin" and "Single-alkane" atmosphere.

| Atmosphere | Temperature (°C) | Conversion (%) | | | | |
|---|---|---|---|---|---|---|
| | | $CH_4$ | $C_2H_6$ | $C_2H_4$ | $C_3H_8$ | $C_3H_6$ |
| $C_3H_8$-$C_3H_6$ | 490 | - | - | - | 33.0 | < 0 |
| $C_3H_8$-$C_2H_4$ | 490 | - | - | 4.3 | 12.3 | - |
| Single- $C_3H_8$ | 490 | - | - | - | 9.4 | - |
| $C_2H_6$-$C_3H_6$ | 520 | - | 8.0 | - | - | 21.5 |
| $C_2H_6$-$C_2H_4$ | 520 | - | 5.0 | < 0 | - | - |
| Single- $C_2H_6$ | | - | 0.9 | - | - | - |
| $CH_4$-$C_2H_4$ | 550 | 7.9 | - | 25.1 | - | - |
| Single- $CH_4$ | | ~0 | - | - | - | - |

$F_{total}$ = 40 mL $min^{-1}$, "Single-Alkane", Alkane: $O_2$: $N_2$ = 8: 8: 24; "Alkane-Olefin", Alkane: Olefin: $O_2$: $N_2$ = 8: 2.5: 8: 21.5, $m_{cat}$ = 100 mg.

**Supplementary Table 2** Specific treatments of the catalysts.

| Catalysts | Treatments |
|---|---|
| fresh BN | - |
| activated BN | $F_{total}$ = 40 mL $min^{-1}$, $C_3H_8$: $O_2$: $N_2$ = 8: 8: 24, 550 °C, 3 h |
| Single-$C_3H_8$-BN | Process 1: $F_{total}$ = 40 mL $min^{-1}$, $C_3H_8$: $O_2$: $N_2$ = 8: 8: 24, 550 °C, 3 h<br>Process 2: $F_{total}$ = 40 mL $min^{-1}$, $C_3H_8$: $O_2$: $N_2$ = 8: 8: 24, 500 °C, 3 h |
| $C_3H_8$-$C_3H_6$-BN | Process 1: $F_{total}$ = 40 mL $min^{-1}$, $C_3H_8$: $O_2$: $N_2$ = 8: 8: 24, 550 °C, 3 h<br>Process 2: $F_{total}$ = 40 mL $min^{-1}$, $C_3H_8$: $C_3H_6$: $O_2$: $N_2$ = 8: 2.5: 8: 24, 500 °C, 3 h |

**Supplementary Table 3** Alkane conversions, products distribution, and space-time yield at similar alkane conversions under different feeding modes.

| Mode | weight hourly space velocity (WHSV, g $g^{-1}$ $h^{-1}$) | | | Conversion (%) | | | Selectivity (%) | | | | Space-time yield (mmol $g_{cat}^{-1}$ $h^{-1}$) | | |
|---|---|---|---|---|---|---|---|---|---|---|---|---|---|
| | $C_2H_6$ | $C_3H_8$ | Alkanes | $C_2H_6$ | $C_3H_8$ | Alkanes | $C_2H_4$ | $C_3H_6$ | CO | Olefins | $C_2H_4$ | $C_3H_6$ | Olefins |
| $C_2H_6$-$C_3H_8$ (510 °C) | 6.0 | 2.9 | 8.9 | 4.0 | 8.0 | 5.2 | 57.0 | 42.6 | 0.4 | 99.6 | 9.3 | 4.6 | 13.9 |
| Single- $C_2H_6$ (540 °C) | 6.0 | 0 | 6.0 | 3.9 | 0 | 3.9 | 100 | 0 | 0 | 100 | 8.2 | 0 | 8.2 |
| Single- $C_3H_8$ (530 °C) | 0 | 2.9 | 2.9 | 0 | 8.5 | 8.5 | 10.4 | 89.0 | 0.6 | 99.4 | 0.9 | 5.1 | 6.0 |
| “Single- $C_2H_6$” + “Single- $C_2H_8$” | 6.0 | 2.9 | 8.9 | 3.9 | 8.5 | 5.3 | 54.4 | 45.3 | 0.3 | 99.7 | 9.1 | 5.1 | 14.2 |

$F_{total}$ = 40 mL $min^{-1}$, “Single-$C_2H_6$”, $C_2H_6$: $O_2$: $N_2$ = 8: 8: 24; “Single-$C_3H_8$”, $C_2H_6$: $O_2$: $N_2$ = 2.5: 8: 29.5;

“$C_2H_6$-$C_3H_8$”, Alkane: Olefin: $O_2$: $N_2$ = 8: 2.5: 8: 21.5, $m_{cat}$ = 100 mg.

## Supplementary References

1. Sainsbury, T. *et al.* Oxygen Radical Functionalization of Boron Nitride Nanosheets. *J. Am. Chem. Soc.* **134**, 18758–18771 (2012).

2. Grant, J. T. *et al.* Selective oxidative dehydrogenation of propane to propene using boron nitride catalysts. *Science (80-. ).* **354**, 1570–1573 (2016).

3. Huang, R. *et al.* Direct Insight into Ethane Oxidative Dehydrogenation over Boron Nitrides. *ChemCatChem* **9**, 3293–3297 (2017).

4. Liu, Z. *et al.* Plasma Tuning Local Environment of Hexagonal Boron Nitride for Oxidative Dehydrogenation of Propane. *Angew. Chemie Int. Ed.* **60**, 19691–19695 (2021).

5. Zhao, Z. J. *et al.* Hydroxyl-Mediated Non-oxidative Propane Dehydrogenation over VOx/γ-Al2O3 Catalysts with Improved Stability. *Angew. Chemie - Int. Ed.* **57**, 6791–6795 (2018).
